# D‐ and L‐Amino Acid Blood Concentrations Are Affected in Children With Duchenne Muscular Dystrophy

**DOI:** 10.1111/jcmm.70495

**Published:** 2025-05-06

**Authors:** Martina Garofalo, Chiara Panicucci, Alberto Imarisio, Tommaso Nuzzo, Noemi Brolatti, Maria Egle De Stefano, Enza Maria Valente, Francesco Errico, Claudio Bruno, Alessandro Usiello

**Affiliations:** ^1^ Department of Environmental, Biological and Pharmaceutical Sciences and Technologies Università Degli Studi Della Campania “Luigi Vanvitelli” Caserta Italy; ^2^ CEINGE Biotecnologie Avanzate Franco Salvatore Naples Italy; ^3^ Centre of Translational and Experimental Myology IRCCS Istituto Giannina Gaslini Genoa Italy; ^4^ Department of Molecular Medicine University of Pavia Pavia Italy; ^5^ Neurogenetics Research Centre IRCCS Mondino Foundation Pavia Italy; ^6^ Department of Biology and Biotechnologies “Charles Darwin” Sapienza University Rome Italy; ^7^ Department of Agricultural Sciences University of Naples “Federico II” Portici Italy; ^8^ Department of Neuroscience, Rehabilitation, Ophthalmology, Genetics, Maternal and Child Health‐DINOGMI University of Genoa Genoa Italy

**Keywords:** amino acids, biomarker, D‐aspartate, D‐serine, Duchenne muscular dystrophy, glutamate, motor dysfunction, muscle wasting, serum

## Abstract

Duchenne muscular dystrophy (DMD) is an X‐linked disease caused by the absence of functional dystrophin in the muscle cells. Recent untargeted metabolomics studies identified amino acid metabolism alterations as biochemical pathways potentially involved in DMD pathogenesis. Here, in a well‐characterised cohort of DMD children and paediatric controls, we investigated by high‐performance liquid chromatography (HPLC) the serum profile of a selected pool of amino acids in D‐ and L‐configuration, including L‐glutamate, L‐glutamine, glycine, L‐aspartate, D‐aspartate, L‐asparagine, L‐serine, and D‐serine. These amino acids are known to modulate neurotransmission and to play essential roles in energy and skeletal muscle metabolism. HPLC determinations highlighted a general amino acid deregulation in DMD compared to controls, including lower levels of L‐aspartate, L‐asparagine, D‐serine, L‐glutamine, and glycine and D−/Total serine ratio. In control subjects, we observed a significant positive correlation between L‐glutamine and age, which lacked in affected children. Conversely, in DMD, we observed (i) a negative correlation of L‐glutamate and L‐aspartate with serum creatinine and creatine kinase levels; (ii) a direct correlation of serum L‐glutamine/L‐glutamate ratio with the fat‐free mass index (as determined by dual energy X‐ray absorptiometry) and with specific motor function scores (North Star Ambulatory Assessment); and (iii) no correlations between glucocorticoid treatment or cognitive function and the serum amino acid profile. Our study highlights significant correlations between serum L‐glutamate levels, L‐glutamine/L‐glutamate ratio, and the multidimensional measures of muscle wasting and motor impairment, suggesting that peripheral glutamine‐glutamate metabolism can be a suitable biomarker of disease severity and progression in DMD patients.

**Abbreviations**
AD: Alzheimer's disease
ADHD: attention deficit hyperactivity disorder
BMI: body mass index
CK: creatine kinase
DMD: Duchenne muscular dystrophy
DXA: dual energy X‐ray absorptiometry
FEV: forced expiratory volume
FFMI: fat‐free mass index
FMI: fat mass index
FVC: forced vital capacity
HPLC: high‐performance liquid chromatography
IQ: intelligence quotient
NMDAR: N‐methyl‐D‐aspartate glutamate receptor
NSAA: North Star Ambulatory Assessment
PCF: peak cough flow

## 1 Background

Duchenne muscular dystrophy (DMD) is a recessive X‐linked disorder caused by pathogenic variants in the DMD gene, which encodes the dystrophin protein. Dystrophin deficiency leads to fibre degeneration and induces dystrophic changes at the muscle level, resulting in progressive muscular weakness [1]. Although histologic and laboratory evidence of myopathy may be observed from birth among male children with DMD, the clinical onset of weakness usually occurs within three years of age. An unusual waddling gait, lumbar lordosis, and calf pseudohypertrophy are often observed [2]. DMD‐affected children present a variable degree of cognitive and learning disability, ranging from normal cognition to a global developmental delay, and up to one third experience attention deficit hyperactivity disorder (ADHD) [3]. The prevalence of DMD in the general Italian population is 1.7–3.4 cases per 100,000 [4], which is in line with the estimated global DMD prevalence [5].

Although several approaches, including micro‐dystrophin gene therapy and treatment with exon skipping‐inducing antisense oligonucleotides, have been approved or are currently under approval [6, 7, 8], glucocorticoids remain the gold standard treatment for all DMD patients, with proven potential to slow disease progression [9]. Non‐linear disease progression, high phenotypic variability related to several genetic modifiers, the rarity of the disease, and variability in the standard of care represent unsolved hurdles that are currently hampering the development of innovative DMD therapies in clinical trials. At present, available biomarkers of disease severity and progression have several limitations, including reproducibility between different centres and operator dependence (muscle magnetic resonance imaging), patient compliance (functional outcomes), and low specificity (serum creatine kinase (CK) levels) [9, 10]. These limitations make the identification of new, accessible, and reliable prognostic and predictive biomarkers for DMD an urgent unmet clinical need.

Untargeted serum and skeletal muscle metabolomics studies in DMD murine models and DMD patients showed extensive alterations in lipid, energy, and nitric oxide metabolism, as well as activation of inflammatory pathways in DMD compared to controls [11, 12, 13, 14, 15, 16, 17, 18]. Moreover, serum amino acid metabolism dysregulation was identified as a biochemical signature potentially involved in disease pathogenesis [16, 18, 19]. However, previous OMICS results are inconsistent among independent studies [10] and, except for serum creatine and creatinine levels, which are respectively increased and decreased in DMD [17, 18], reliable biochemical markers representative of DMD are currently lacking. In light of this knowledge gap, here, we measured by high‐performance liquid chromatography (HPLC) in the serum of a well‐characterised cohort of DMD patients and paediatric controls without neuromuscular disorders, a selected pool of amino acids either acting on glutamatergic receptors activation (L‐glutamate, L‐aspartate, D‐aspartate, glycine, and D‐serine) or representing the immediate precursors of these neuroactive molecules (L‐glutamine, L‐asparagine, and L‐serine). Despite their “atypical” D‐configuration, both D‐aspartate and D‐serine occur at significant levels in mammalian tissues, especially in the central nervous system, where they act as agonist and co‐agonist of the NMDA subtype of glutamate receptors (NMDARs), respectively [20, 21].

In addition to their neuroactive role, all the detected amino acids play key roles in regulating various cellular pathways, including protein synthesis (only L‐stereoisomers), tricarboxylic acid cycle, redox homeostasis, ammonium recycling, purine nucleotide cycle, folate and methionine cycles, hormone biosynthesis and release, and phospholipids biosynthesis [22]. Given their relevance in modulating the metabolism of the skeletal muscles, which represents the main system affected in DMD patients, we hypothesised that the serum level of these amino acids may be altered in DMD compared to subjects without neuromuscular disorders. Finally, we investigated the relationship between these biomolecules and the clinical‐biochemical features of DMD, including markers of muscle mass, motor, respiratory and cognitive functions, and body composition.

## 2 Methods

### 2.1 Participants

This observational, cross‐sectional study included 29 genetically diagnosed DMD patients referred to the IRCCS Istituto Giannina Gaslini in Genova. On the day of assessment, anamnestic data, such as steroid treatment duration, ambulatory status (i.e., ambulant or not ambulant), and the need for non‐invasive ventilation (NIV), were collected by charts review, and anthropometric parameters (height, weight and BMI) were measured. BMI SD was calculated according to the Italian growth charts [23]. Measures of motor performance and pulmonary function tests were conducted by trained clinical evaluators. Specifically, ambulatory patients underwent the North Star Ambulatory Assessment (NSAA). forced vital capacity (FVC), forced expiratory volume (FEV), and peak cough flow (PCF) were reported in both absolute values and fractions (litres, litres per minute, %). PCF was defined as abnormal if less than 270 L/min.

A formal cognitive assessment was conducted by a psychologist in a patients' subgroup (*n* = 18), applying different neuropsychological scales tailored to the participants' age groups. Specifically, the Wechsler Preschool and Primary Scale of Intelligence‐III (WPPSI‐III) was administered to subjects aged between 2 and 6 years; the Wechsler Intelligence Scale for Children‐IV (WISC‐IV) was used for boys aged between 6 years and 16 years and 11 months; and the Wechsler Adult Intelligence Scale‐IV (WAIS‐IV) was used for patients over 16 years and 11 months. Each scale comprises multiple subtests, allowing the calculation of an overall score (intelligence quotient, IQ). Intellectual disability was defined for IQ values < 70, in accordance with the Diagnostic and Statistical Manual of Mental Disorders, fourth edition, text revision (DSM IV‐TR). For the remaining eleven patients, neuropsychological scales could not be conducted due to logistical reasons. In such cases, the assessment of cognitive aspects relied on their school performance: patients who could attend school without the aid of a support teacher were categorised into the “normal cognition” group, while those who required assistance were classified into the “impaired cognition” group.

Total body dual energy X‐ray absorptiometry (DXA) measurements (Lunar Prodigy, GE) were conducted to obtain body composition measures such as fat mass (FM) and fat‐free mass (FFM) as absolute values and fractions (kg, %). To represent the proportion of fat contributing to the BMI value, the fat mass index (FMI) was calculated as [FM by DXA in kg/(height in meters)^2^]. The fat‐free mass index (FFMI) was calculated as [FFM by DXA in kg/(height in meters)^2^].

Twenty‐four age‐ and sex‐matched controls were recruited for the study, each contributing a single blood sample. These participants were patients referred to other units for various conditions, none of whom had any of the following exclusion criteria: (i) diagnosis of neuromuscular disorder, (ii) elevated CK levels, and (iii) receiving intramuscular therapies (Table S1).

### 2.2 Collection and Storage of Serum Samples

Blood sampling was performed after a 12‐h fast. Whole blood was collected by peripheral venipuncture into clot activator tubes and gently mixed. The sample was stored upright for 30 min at room temperature to allow the blood to clot and centrifuged at 2000 × g for 10 min at room temperature. The serum was aliquoted (0.5 mL) in polypropylene cryotubes and stored at −80°C before usage. Blood samples were processed according to our previously validated protocol [24, 25].

### 2.3 HPLC Analysis of Serum Amino Acid Content

Blood serum samples were analysed as previously reported [24, 26]. Serum samples (100 μL) were mixed in a 1:10 dilution with HPLC‐grade methanol (900 μL) and centrifuged at 13,000×g for 10 min; supernatants were dried and then suspended in 0.2 M trichloroacetic acid (TCA). TCA supernatants were then neutralised with 0.2 M NaOH and subjected to precolumn derivatisation with o‐phthaldialdehyde/N‐acetyl‐L‐cysteine in 50% methanol. Amino acid derivatives were resolved on a ZORBAX Eclipse Plus C8 5‐μm reversed‐phase column (Agilent, 4.6×250 mm) under isocratic conditions (0.1 M sodium acetate buffer, pH 6.2, 1% tetrahydrofuran, 1 mL/min flow rate). A washing step in 0.1 M sodium acetate buffer, 3% tetrahydrofuran, and 47% acetonitrile was performed after every run. Identification and quantification of amino acids were based on retention times and peak areas, compared with those associated with external standards. The identity of the D‐Asp peak was further evaluated by selective degradation catalysed by a recombinant human D‐aspartate oxidase enzyme (hDDO) [27]. hDDO enzyme (~ 12 μg) was added to the samples, incubated at 30°C for 3 h, and subsequently derivatised. Each HPLC experiment was performed in duplicate. The detected amino acid concentration was expressed as μM, D−/total serine ratio as percentage, and glycine/L‐serine ratio as an absolute number. Researchers who performed the HPLC analyses were blinded to the clinical status of participants.

### 2.4 Statistical Analyses

Clinical and demographic characteristics were described using, as summary statistics, the median and the interquartile range (IQR) or absolute and relative frequencies. The comparison of clinical‐demographic features between DMD and control groups was performed with the Mann–Whitney U test for continuous variables and the Chi‐square test for categorical variables. Fisher's exact test was used when at least one expected frequency in a fourfold table was less than five. The correlation between serum amino acids concentrations and clinical‐demographic features was evaluated with Spearman's correlation test. The *p*‐values obtained from correlation tests were also adjusted with the Benjamini–Hochberg method (false discovery rate, FDR) to account for multiple testing [28]. FDR *q*‐value < 0.05 was considered significant. Given the exploratory nature of the study, we considered of interest also the results that did not survive the FDR correction; however, the correlation coefficients that remained significant after FDR correction are highlighted within the Tables 3 and 4. All the statistical tests were two‐tailed. Data were analysed by using SPSS 26.0 software (IBM, Armonk, NY, USA).

## 3 Results

### 3.1 Serum Amino Acids Profile Is Disrupted in DMD Patients

**FIGURE 1:**
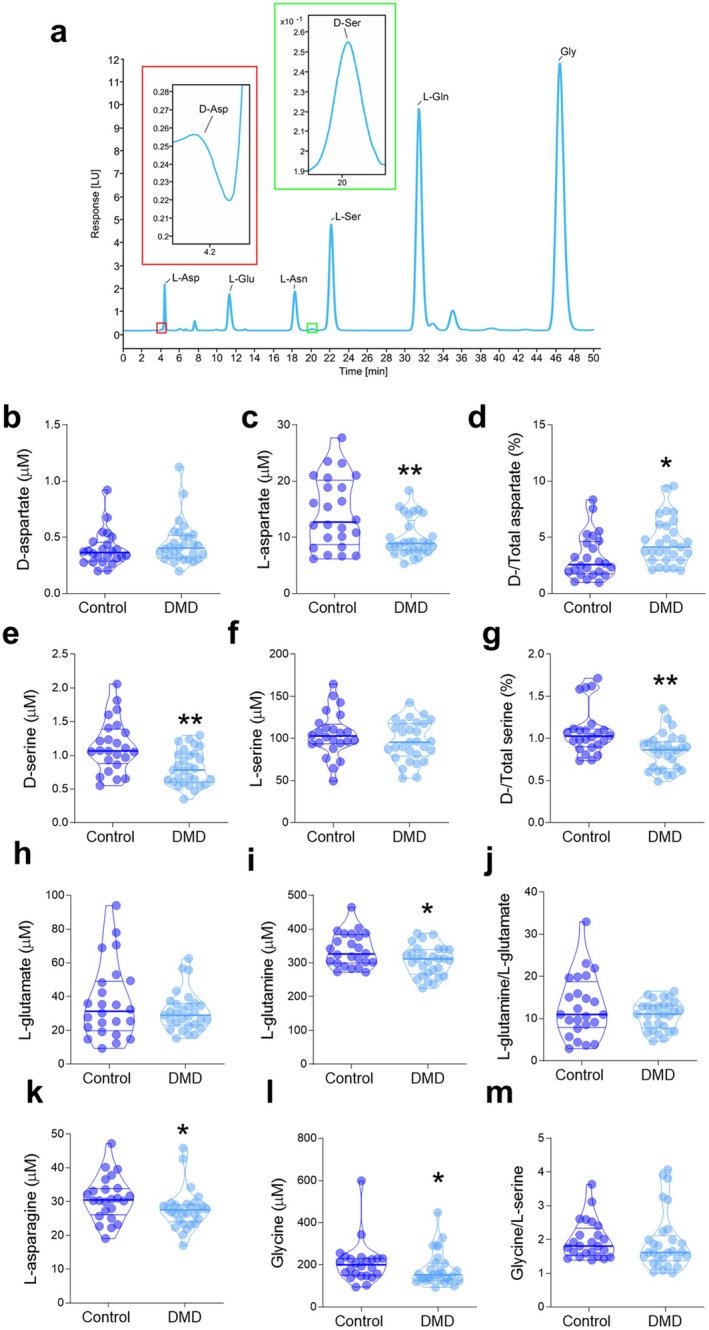
(a) Representative HPLC chromatogram illustrating the amino acids peaks obtained from a serum sample; (b–m) Violin plots showing serum amino acid levels in DMD patients (*n* = 29) and controls (*n* = 24). Data are shown as median (bold line), 25°–75° percentiles (inner lines), min and max values (lower and upper violin extremities). *p < 0.05; **p < 0.01, Mann–Whitney U test.

Twenty‐nine DMD patients and twenty‐four controls were enrolled in the study. The demographic and clinical features of the groups are reported in Table 1. We first investigated the serum levels of amino acids in DMD patients and control subjects. DMD patients showed comparable D‐Asp content compared to controls (*p* = 0.231; Figure 1b) but decreased levels of L‐aspartate (*p* = 0.010; Figure 1c and Table 2) and its precursor L‐asparagine (*p* = 0.018; Figure 1k and Table 2). Consequently, the D‐aspartate to total aspartate ratio (D/D + L) was increased in DMD patients compared to controls (*p* = 0.011; Figure 1d and Table 2). Moreover, HPLC data showed reduced concentration of D‐serine and D−/total serine ratio in DMD compared to controls (D‐serine: *p* = 0.001; D−/total serine, *p* = 0.001; Figure 1e,g and Table 2), while L‐serine levels were unaltered between groups (*p* = 0.30; Figure 1f and Table 2). Glycine concentration was decreased in DMD patients compared to controls (*p* = 0.043; Figure 1l and Table 2), while glycine/L‐serine ratio was not affected (*p* = 0.025; Figure 1m and Table 2). Furthermore, we reported lower L‐glutamine content in DMD subjects (*p* = 0.04; Figure 1i and Table 2) along with unaltered L‐glutamate and L‐glutamine/L‐glutamate ratio (L‐glutamate, *p* = 0.72; L‐glutamine/L‐glutamate *p* = 0.63; Figure 1h,j and Table 2).

**TABLE 1:** Demographic and clinical features of DMD patients and controls.

	*n*	DMD	Controls	*p*
Age (years)	24 C, 29 DMD	10.2 (5.0–15.7)	9.8 (5.9–14.3)	0.830^a^
Male sex, *n* (%)	24 C, 29 DMD	29 (100)	24 (100.0)	—
BMI (kg/m^2^)	20 C, 29 DMD	18.3 (16.1–20.2)	16.9 (15.1–21.0)	0.339^a^
Standardised BMI	20 C, 29 DMD	−0.1 (−1.2–0.9)	−0.4 (−1.2–0.7)	0.640^a^
Creatinine (mg/dl)	20 C, 29 DMD	0.2 (0.1–0.2)	0.5 (0.4–0.6)	**< 0.001** ^a^
CK (UI/l)	5 C, 29 DMD	8919 (3276–16,293)	114 (80–154)	**< 0.001** ^a^
Fasting glycemia (mg/dl)	21 C, 29 DMD	92.0 (87.5–96.0)	93 (86–103)	0.503^a^
Able to walk, *n* (%)	24 C, 29 DMD	22 (75.9)	24 (100.0)	**0.012** ^b^
Need for ventilator support, *n* (%)	29 DMD	4 (13.8)	—	—
Intellectual disability, *n* (%)	29 DMD	10 (34.5)	—	—
IQ	18 DMD	95 (80–100)*	—	—
Fat mass (kg)	17 DMD	13.7 (7.6–21.2)	—	—
FMI (kg/m^2^)	17 DMD	6.2 (4–6‐9.4)	—	—
Fat mass (%)	17 DMD	37.3 (29.8–48.8)	—	—
Fat‐free mass (kg)	17 DMD	20.9 (13.8–24.7)	—	—
FFMI (kg/m^2^)	17 DMD	11.0 (9.5–12.5)	—	—
Systemic steroid treatment at blood sampling, *n* (%)	29 DMD	17 (58.6)	—	—
Steroid treatment duration (years)	17 DMD	4.3 (2.3–10.0)	—	—
NSAA	15 DMD	20.0 (13.0–27.0)	—	—
FVC (l)	18 DMD	1.65 (1.13–2.03)	—	—
FVC (%)	18 DMD	82.5 (66.5–94.5)	—	—
FEV1 (l)	18 DMD	1.36 (1.07–1.93)	—	—
FEV1 (%)	18 DMD	94.0 (74.2–102.5)	—	—
PCF (l/min)	18 DMD	153.6 (88.2–253.2)	—	—
PCF abnormal, *n* (%)	18 DMD	15 (83.3)	—	—

*Note:* Data are shown as median (IQR). The number of subjects for which data were available is shown in the second column.

Abbreviations: BMI, body mass index; CK, creatine kinase; FEV1, forced expiratory volume in the first second; FFMI, fat‐free mass index; FMI, fat mass index; FVC, forced vital capacity; IQ, intelligence quotient; NSAA, North Star ambulatory assessment; PCF, peak cough flow.

^a^ Mann–Whitney U test.

^b^ Fisher exact test.

**TABLE 2:** Serum amino acid levels in DMD patients and controls.

	DMD (*n* = 29)	Controls (*n* = 24)	*p* ^a^
D‐aspartate (μM)	0.4 (0.3–0.5)	0.4 (0.3–0.5)	0.231
L‐Aspartate (μM)	8.9 (7.6–13.0)	12.7 (8.6–20.1)	**0.010**
D−/Total aspartate (%)	4.1 (2.9–6.0)	2.6 (1.7–4.6)	**0.011**
L‐asparagine (μM)	27.5 (24.0–29.1)	30.4 (26.1–33.8)	**0.018**
L‐glutamate (μM)	29.0 (23.1–35.8)	31.2 (19.7–49.1)	0.721
L‐glutamine (μM)	311.4 (266.4–339.8)	326.0 (299.3–383.7)	**0.040**
L‐glutamine/L‐glutamate	11.0 (7.6–13.0)	10.9 (7.8–18.7)	0.629
D‐serine (μM)	0.8 (0.6–1.0)	1.1 (0.9–1.4)	**0.001**
L‐serine (μM)	95.5 (79.1–117.5)	102.8 (93.7–116.8)	0.300
D−/Total serine (%)	0.9 (0.6–1.0)	1.0 (0.9–1.1)	**0.001**
Glycine (μM)	152.4 (127.0–208.3)	200.3 (150.5–229.7)	**0.043**
Glycine/L‐serine	1.6 (1.3–2.1)	1.8 (1.5–2.3)	0.245

*Note:* Data are shown as median (IQR). Significant p‐values are shown in bold.

^a^ Mann–Whitney U test.

These findings implicate that the serum profile of several amino acids is affected in DMD. The altered levels of D−/total serine and D−/total aspartate ratios, which represent reliable indexes of D‐serine and D‐aspartate metabolism [20, 29, 30, 31, 32], suggest that DMD may also affect the D‐serine vs. L‐serine and D‐aspartate vs. L‐aspartate interconversion homeostasis at the systemic level.

### 3.2 Increased Serum L‐Glutamate Levels and D−/Total Serine Ratio Mirror Muscle Loss in DMD Patients

Next, we investigated the relationship between the serum amino acid levels and clinical –demographic features in the DMD and control groups.

First, we performed correlation analyses of serum amino acid levels with age. Spearman's correlation analysis showed a significant positive correlation between L‐glutamine and age in controls (Table 3), while no correlations between the serum amino acid concentrations and age occurred in DMD patients (Table 4). We then evaluated the correlations between serum amino acids and body composition measures. We reported no significant correlation with BMI in DMD and control groups, except for a negative correlation between L‐serine and BMI in DMD patients (Tables 3 and 4). Interestingly, in DMD children, the levels of L‐glutamate and glycine, along with the glycine/L‐serine ratio, correlated positively with the percentage of fat mass and FMI (Table 4); however, only the associations between the glycine/serine ratio and fat mass parameters survived the FDR adjustment (Table 4). Moreover, we found that L‐glutamate correlated negatively and L‐glutamine/L‐glutamate positively with the amount of fat‐free mass expressed as FFMI, but these correlations were not significant after FDR correction.

**TABLE 3:** Correlation between serum amino acid levels and clinical‐demographic features in controls.

	D‐Asp	L‐Asp	D−/Total Asp	L‐Asn	L‐Glu	L‐Gln	L‐Gln/L‐Glu	D‐Ser	L‐Ser	D−/Total Ser	Gly	Gly/L‐Ser
Age (years)	−0.078	−0.023	−0.031	0.339	−0.140	**0.602** ^a^	0.270	0.117	0.333	−0.026	0.030	0.040
Std BMI (kg/m^2^)	0.135	0.195	−0.062	−0.205	0.293	0.057	−0.289	−0.068	−0.165	0.098	0.053	0.084
Creatinine (mg/dl)	0.233	0.105	0.062	0.434	−0.117	**0.564**	0.281	0.273	**0.473**	0.086	0.092	−0.174
Fasting glycemia (mg/dl)	−0.181	0.016	−0.005	0.366	0.072	0.200	0.010	0.402	0.398	0.012	0.306	0.167

*Note:* Spearman's correlation coefficients are reported. Only clinical features available in at least *n* = 10 subjects were considered for analyses. Bold indicates significant correlations at *p* < 0.05.

Abbreviation: Std BMI, standardised body mass index.

^a^ Significant after FDR correction.

**TABLE 4:** Correlation between serum amino acid levels and clinical‐demographic features in DMD patients.

	D‐Asp	L‐Asp	D−/Total Asp	L‐Asn	L‐Glu	L‐Gln	L‐Gln/L‐Glu	D‐Ser	L‐Ser	D−/Total Ser	Gly	Gly/L‐Ser
Age (years)	−0.123	0.265	−0.259	0.145	0.292	0.365	−0.251	0.096	0.314	−0.189	0.305	0.067
Std BMI (kg/m^2^)	−0.082	−0.158	0.133	0.043	−0.194	−0.179	0.200	−0.137	**−0.380**	0.286	−0.035	0.173
Creatinine (mg/dl)	−0.072	**−0.442** ^a^	0.247	0.347	**−0.559** ^a^	0.159	**0.684** ^a^	0.297	−0.246	**0.666** ^a^	−0.292	−0.142
CK (UI/l)	0.156	**−0.389**	0.340	0.050	**−0.445**	−0.118	**0.486**	0.024	−0.352	**0.379**	−0.209	0.008
Fasting glycemia (mg/dl)	−0.113	−0.016	−0.065	0.192	0.276	0.077	−0.245	−0.088	0.228	**−0.368**	0.103	−0.070
Steroid treatment duration (years)	0.259	0.343	0.056	0.199	0.267	0.453	−0.186	0.179	0.311	−0.027	0.475	0.282
IQ	0.252	0.081	0.105	0.138	−0.039	0.110	−0.052	0.293	0.357	−0.124	−0.258	−0.438
FMI (kg/m^2^)	−0.010	0.370	−0.236	−0.196	**0.525**	0.206	−0.401	−0.363	−0.286	0.007	**0.578**	**0.658** ^a^
Fat mass (%)	0.002	0.373	−0.270	−0.336	**0.574**	0.078	**−0.534**	−0.431	−0.289	−0.071	**0.561**	**0.667** ^a^
FFMI (kg/m^2^)	0.137	−0.304	0.294	0.329	**−0.493**	0.259	**0.625**	0.301	−0.007	0.393	−0.283	−0.317
NSAA	0.318	−0.202	0.136	−0.054	−0.326	0.277	**0.549**	−0.039	−0.193	0.131	−0.066	0.191
FVC (%)	−0.089	−0.433	0.256	0.138	−0.267	−0.138	0.371	0.054	−0.324	0.324	−0.443	−0.148
FEV1 (%)	−0.176	−0.416	0.146	0.092	−0.307	−0.207	0.337	0.067	−0.387	0.437	−0.344	−0.038
PCF (l/min)	0.019	−0.102	0.183	**0.531**	0.024	0.439	0.269	0.315	0.112	0.205	−0.166	−0.069

*Note:* Spearman's correlation coefficients are reported. Only clinical features available in at least *n* = 10 patients were considered for analyses. Bold indicates significant correlations at *p* < 0.05.

Abbreviations: CK, creatine kinase; FEV1, forced expiratory volume in the first second; FFMI, fat‐free mass index; FMI, fat mass index; FVC, forced vital capacity; IQ, intelligence quotient; NSAA, North Star ambulatory assessment; PCF, peak cough flow; Std BMI, standardised body mass index.

^a^ Significant after FDR correction.

**FIGURE 2:**
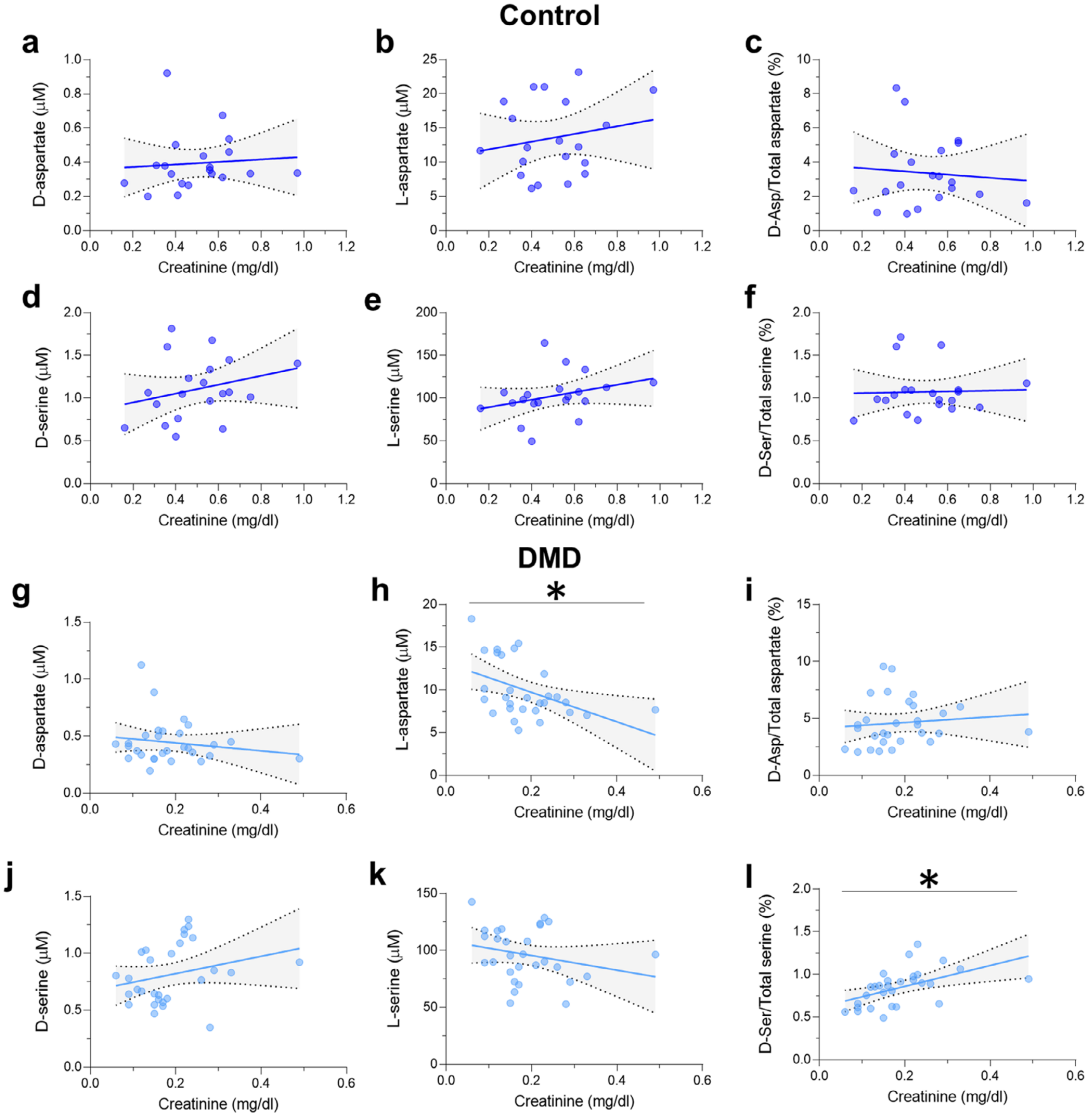
Correlations between serum D‐aspartate, L‐aspartate, D−/Total aspartate ratio, D‐serine, L‐serine, D−/Total serine ratio and creatinine in DMD (*n* = 29) and control (*n* = 20) groups. Blue lines and grey shadows represent the best fit line and its 95% CI, respectively. *q < 0.05; Spearman's correlation test with FDR correction.

**FIGURE 3:**
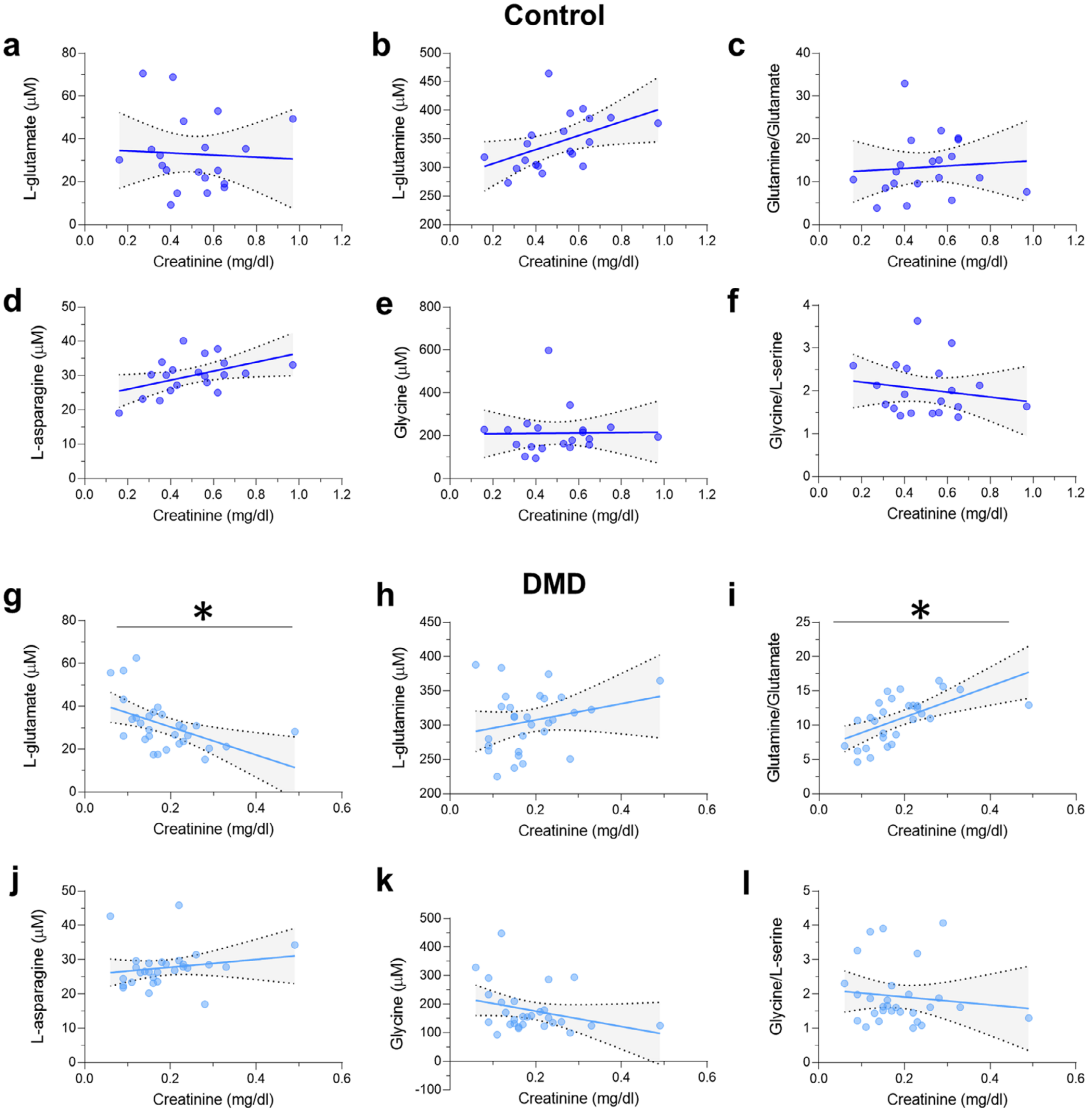
Correlations between serum L‐glutamate, L‐glutamine, L‐glutamine/L‐glutamate ratio, L‐asparagine, glycine, glycine/L‐serine ratio and creatinine in DMD (*n* = 29) and control (*n* = 20) groups. Blue lines and grey shadows represent the best fit line and its 95% CI, respectively. *q < 0.05; Spearman's correlation test with FDR correction.

We then evaluated the association between serum amino acid levels and creatinine or CK levels. In the control group, L‐serine and L‐glutamine positively correlated with serum creatinine; however, these correlations were not significant after adjusting for multiple testing (Figure 2e, Figure 3b and Table 3). All the other amino acids were not significantly correlated with the creatinine levels (Figures 2 and 3 and Table 3). In the DMD group, L‐glutamate and L‐aspartate correlated negatively with serum creatinine levels (Figure 2g, Figure 3h and Table 4), while L‐glutamine/L‐glutamate and D−/total serine ratios correlated positively with creatinine (Figure 2l, Figure 3i and Table 4). Of note, these correlations remained significant even after FDR correction (*q*‐value < 0.05) (Table 4 and Figures 2 and 3). Moreover, in DMD patients, serum CK levels correlated negatively with L‐glutamate and L‐aspartate and positively with L‐glutamine/L‐glutamate and D−/total serine ratios (Table 4); however, these correlations did not survive FDR adjustment (*q*‐value > 0.05).

Next, we evaluated the associations between amino acid levels and the clinical features of DMD patients. The serum amino acid profile was not different between patients treated or nottreated with systemic corticosteroids at the time of blood sampling (Table S2). Moreover, there were no correlations between the duration of treatment with deflazacort and the amino acid concentration (Table 4). There were no associations between the serum amino acid profile and the measures of motor performance (NSAA) and pulmonary function tests, except for positive correlations between L‐glutamine/L‐glutamate and NSAA, and between L‐asparagine and PCF (Table 4), which did not survive correction for multiple testing. Non‐ambulant patients showed increased serum L‐aspartate, L‐glutamate, L‐serine, and glycine levels and lower L‐glutamine/L‐glutamate and D−/total serine ratios compared to ambulant children (Table S3).

We did not find any significant association between the amino acid concentration and IQ or dichotomic (normal vs. impaired) cognitive status (Table 4 and Table S4).

Overall, these findings indicate that the serum profile of amino acids in DMD is mainly associated with measures of muscle mass, especially creatinine. L‐aspartate, L‐glutamate, and D−/total serine ratio may thus serve as surrogate markers of muscular wasting in DMD.

## 4 Discussion

In this study, we found that the serum concentration of various amino acids, including L‐aspartate, L‐asparagine, D‐serine, L‐glutamine, and glycine, was down‐regulated in DMD compared to controls. Moreover, L‐aspartate and L‐glutamate correlated negatively with serum creatinine and CK levels, while L‐glutamine/L‐glutamate and D−/total serine correlated positively with these biochemical measures of muscle wasting.

Our findings, showing a down‐regulated serum profile of several amino acids in DMD children, are in line with previous metabolomics studies which reported decreased serum levels of L‐aspartate [18], L‐glutamate [16], and L‐glutamine [16, 19] in DMD compared to controls. Consistent with this data, pathway enrichment analyses showed an extensive dysregulation in the serum metabolism of the urea cycle and amino groups [17], alanine/aspartate [18] and aspartate, glutamine, and glutamate pathways [16] in DMD patients. Overall, these findings confirm a complex derangement of amino acid metabolism in DMD and support the hypothesis that altered homeostasis of these vital biomolecules represents a key physiopathological feature of dystrophinopathies.

Interestingly, a previous targeted biochemical study failed to find any significant difference in the plasma concentration of 33 amino acids between DMD children and healthy subjects [33]. However, this study was conducted in the pre‐genetic era, when the diagnosis of DMD was based only on the clinical phenotype and muscle histopathological features, thus questioning the actual attribution of the study findings to dystrophinopathies. A previous proton magnetic resonance spectroscopy (H1‐MRS) study conducted on selected brain areas revealed a slight (−7%) decrease in glutamate concentration in the temporo‐parietal region of DMD children compared to healthy controls [34]. In light of the minor entity of the changes observed in the mentioned study, we hypothesise that the reduction in serum L‐glutamate levels observed in our DMD cohort may originate from an altered amino acid metabolism in the peripheral organs rather than in the central nervous system (CNS).

Aside from the above‐mentioned amino acidic pathways, branched chain amino acids (BCAAs, i.e. L‐leucine, L‐isolelucine and L‐valine) are also known to play a central role in skeletal muscle metabolism, and previous studies demonstrated that BCAAs synthesis in visceral tissues is overactivated in various muscle wasting disorders, for example, sepsis, trauma, or surgery [35]. Consistently, BCAA concentrations were shown to be respectively higher in serum [16, 19] and lower in muscle [36] of DMD patients compared to healthy controls. Being the precursor of nitric oxide (NO), L‐arginine is another amino acid crucial for muscle physiology, mitochondrial function, and energy production [37]. The L‐arginine/nitric oxide synthase (NOS) pathway is known to be affected in dystrophinopathies [38], and several authors found altered levels of L‐arginine pathway metabolites in the serum of DMD [17, 18] and Becker muscular dystrophy (BMD) [39] patients compared to healthy subjects. Of note, no previous studies investigated the blood levels of D‐serine and D‐aspartate and the relationship with their respective L‐stereoisomers in DMD children. These atypical amino acids play a key role in glutamatergic neurotransmission and, along with the other related amino acids analysed in this study, directly modulate mitochondrial function and immune system homeostasis [40, 41], which are both known to be disrupted in DMD [42, 43].

Remarkably, we found a positive correlation between serum L‐glutamine concentration and age in controls but not in DMD children, indicating that the dynamics of peripheral L‐glutamine changes during development may be disrupted in DMD. Moreover, DMD children showed strong negative correlations of L‐aspartate and L‐glutamate with serum creatinine, while L‐glutamine/L‐glutamate and D−/total serine ratios positively correlated with creatinine. Despite non‐surviving FDR correction, a similar trend of correlation was observed between the same amino acids and serum CK level in the DMD group. The creatine and phosphocreatine system plays a crucial role as an energy‐supporting mechanism in skeletal muscle, where creatine is primarily found [44]. Creatine and phosphocreatine in myofibers are non‐enzymatically converted to creatinine at a constant rate, which is then released into the bloodstream. Serum creatinine levels are therefore correlated with muscle mass and have thus been used as a surrogate biomarker for estimating total body muscle mass [45, 46, 47]. Importantly, blood creatinine and CK have also been proposed as potential biomarkers of disease severity and motor progression in muscular dystrophies and in spinal muscular atrophy [48]. Thus, our findings suggest that L‐aspartate, L‐glutamate, and D−/total serine ratio may serve as surrogate biomarkers of disease progression in DMD.

Additionally, the correlation with the DXA body composition measures disclosed a negative correlation of L‐glutamate and a positive correlation of the L‐glutamine/L‐glutamate ratio with FFMI, a well‐validated and non‐invasive quantitative index of total body lean mass which proved to be useful in monitoring muscle wasting in DMD [49]. This finding further supports the view that increased serum L‐glutamate and decreased L‐glutamine/L‐glutamate ratio may be reliable biochemical signatures of muscle wasting in DMD. Importantly, previous metabolomics studies on adult populations without neuromuscular diseases showed a negative correlation between serum L‐glutamate levels and muscle mass and strength [50, 51, 52]. Taken together, these results implicate a dysregulation of peripheral L‐glutamate metabolism across different conditions causing muscle wasting, including dystrophinopathy‐driven muscle loss and aging‐related sarcopenia. In line with this, we found a negative correlation between the L‐glutamine/L‐glutamate ratio and NSAA score, a widely adopted scale to assess functional motor abilities in ambulant DMD children [53]. The lack of a significant correlation between L‐glutamate and NSAA could be due to the low number of subjects for which the NSAA score was available, thus limiting the statistical power of the analysis. Finally, the higher L‐glutamate, L‐aspartate, L‐serine, and glycine levels observed in non‐ambulant compared to ambulant DMD children (Table S3) further support the hypothesis that increased serum concentrations of these L‐amino acids mirror worse muscle damage in DMD.

Moreover, we observed a positive correlation of L‐glutamate, glycine, and glycine/L‐serine ratio with the percentage of body fat mass and the FMI index, which mirrors the total amount of fat tissue in the body normalised for height. The relationship between serum L‐glutamate and FMI is consistent with the findings of our previous study, which showed a positive correlation between serum L‐glutamate, BMI, and visceral adiposity in an elderly cohort comprising both “fit” and frail subjects [32]. Analogous findings were also reported in several studies conducted in both adult and young children cohorts, suggesting that the link between glutamate and adipose tissue exists before the impact of sex hormones on metabolic asset during puberty [54]. Although the pathophysiology underlying elevated levels of glutamate in the context of obesity is still unclear, considering that glutamate signalling modulates the immune system [55] and that increased visceral adipose mass promotes systemic inflammation [56], elevated blood L‐glutamate levels may represent a metabolic signature underpinning the abnormal increase in oxidative stress and inflammation associated with obesity. Of note, altered homeostasis of adipose tissue is common in DMD children as a consequence of reduced mobility and glucocorticoid treatment, with obesity being highly prevalent among DMD patients [57, 58]. Moreover, the involvement of inflammation in the physiopathology of DMD is well documented, with several studies showing extensive chronic inflammatory processes in the muscle tissue obtained from DMD murine models and patients [13, 42, 59, 60]. A recent metabolomics study performed on DMD patients' muscle biopsies showed abnormally increased levels of glutathione [15], a fundamental cellular anti‐oxidant tripeptide enzymatically synthesised from cysteine, L‐glutamate, and glycine. The altered serum L‐glutamate levels observed in this study may thus mirror several physiopathological hallmarks of DMD, including muscle wasting, adipose tissue dysregulation, and chronic inflammation. Despite this hypothesis being highly intriguing, it should be taken cautiously, also considering that (i) the assessment of blood inflammatory markers was not included in the study design, thus preventing any inference linking the serum amino acid levels and the inflammatory profile and (ii) we did not find any relationship between the serum amino acid levels and the presence or duration of systemic steroid treatment in DMD patients.

To our knowledge, this is the first study to investigate the blood levels of D‐serine and D‐aspartate in a paediatric neuromuscular disorder like DMD. In the CNS, D‐serine acts as an endogenous co‐agonist of the ionotropic NMDAR and plays a key role in modulating synaptic plasticity and cognitive functions [61, 62]. In addition, in line with the involvement of dysfunctional NMDAR‐dependent transmission in a wide range of neurodegenerative and psychiatric disorders, altered levels of D‐serine have been observed in selected brain regions, cerebrospinal fluid, and blood of Parkinson's disease [63, 64, 65], Alzheimer's disease (ad) [66, 67], frontotemporal dementia [68], amyotrophic lateral sclerosis [69], schizophrenia [70], and major depression [31]. Less is known about the physiological roles of D‐aspartate. Pharmacologically, it modulates NMDAR‐mediated transmission, and dysregulation of its metabolism occurs in the brain of schizophrenia patients [71, 72] and animal models of autism spectrum disorders [73]. Although the relationship between the blood levels of these atypical D‐amino acids and cognition is still a matter of debate [74], some authors reported an association between serum D‐serine concentration and cognitive function in ad [66, 75, 76], schizophrenia [77, 78] and frail elderly subjects [32] and between serum D‐aspartate and cognitive decline in AD [79]. Since about one third of DMD children exhibit nonprogressive cognitive impairment and/or ADHD [3], we investigated whether the serum concentration of D‐serine, D‐aspartate, and the other amino acids acting on glutamatergic neurotransmission correlated with intellectual disability and IQ in DMD patients. However, we did not find any association between the serum amino acid levels and the cognitive performance of DMD children. This finding, along with the correlations of L‐ and D‐amino acids with the clinical‐biological measures of muscle mass and body composition observed in this study, may implicate that the peripheral amino acid profile in DMD is mainly due to the metabolism of peripheral organs. Further studies correlating the cerebrospinal fluid or brain levels of D‐serine, D‐aspartate, and the other NMDAR‐stimulating amino acids with cognitive functions in DMD are warranted to address this issue. In contrast, in DMD patients, we observed a positive correlation of the serum D−/total serine ratio with serum creatinine and CK levels and a negative correlation of D−/total serine with fasting glycemia. These findings are consistent with previous studies showing that D‐serine is involved in the metabolism of various peripheral organs, including the skeletal muscle [80], pancreatic islet β cells [81], and kidney [82]. Concerning the last organ, it is important to remark that blood D‐serine levels are strongly correlated with the functionality of glomerular filtration and this D‐amino acid has been recently proposed as a novel biomarker of kidney dysfunction in both adult [82, 83, 84] and children populations [85]. Of note, mild kidney injury may represent a complication in children with DMD [86, 87]. Although no manifest kidney disease was evident in the DMD patients included in this study, we cannot exclude that altered serum D−/total serine ratio and its correlation with creatinine and CK levels could be related, at least in part, to a subtle chronic kidney impairment in DMD children. Further studies should evaluate whether the D−/total serine ratio could be a biomarker of kidney dysfunction, muscle wasting, and blood glucose homeostasis in DMD populations.

The overall alterations in the serum levels of different D‐ and L‐amino acids reported in this study suggest the existence of complex dysfunctions in the biochemical pathways responsible for their biosynthesis and degradation. Future studies in preclinical models of the disorder may be crucial to clarify this point and assess whether specific dysfunctional metabolic mechanisms occur in the CNS, where altered neuroactive amino acid levels may directly contribute to DMD phenotypes, including intellectual disability, autism spectrum disorder (ASD), and ADHD [3, 88, 89].

The strengths of our work include (i) the novelty of investigating the whole profile of D‐amino acids acting on glutamatergic transmission and their precursors in the serum of a well‐characterised DMD cohort and (ii) the correlation of serum amino acids with multiple biochemical and clinical features as well as body composition measures. However, we also acknowledge some limitations. First, the relatively small sample size of the included cohort and the cross‐sectional design of the present study did not allow us to infer any causal relationship between the serum changes in amino acid levels and the clinical features observed in our patients. Future multicentric longitudinal studies on larger dystrophinopathy cohorts, including also BMD patients, are warranted to elucidate this issue. Second, some measures (e.g., IQ, DXA parameters, NSAA, respiratory function tests) were available for only a subset of patients, limiting the statistical power of statistical analyses related to these parameters. Additionally, the lack of ASD and ADHD prevalence assessments prevented a comprehensive understanding of the potential implication of amino acid dysmetabolism in the cognitive and neurodevelopmental profile of DMD patients. Further studies correlating the serum levels of D‐serine and the other NMDAR‐stimulating amino acids with cognitive and behavioural profiles in DMD children are thus warranted. Lastly, the measurement of inflammatory blood markers was not included in the study design, thus hampering the opportunity to investigate whether the serum amino acids profile may be related to the systemic pro‐inflammatory changes previously reported in DMD [60].

## 5 Conclusions

This study highlights a downregulation of several serum amino acid levels in DMD patients compared to controls. The correlations observed between serum L‐glutamate levels, L‐glutamine/L‐glutamate ratio, and the multidimensional measures of muscle wasting and motor impairment implicate that serum L‐glutamate may serve as a potential biomarker of disease severity and progression in DMD.

## Author Contributions

**Martina Garofalo:** investigation (lead). **Chiara Panicucci:** data curation (equal), investigation (lead), writing – review and editing (equal). **Alberto Imarisio:** data curation (equal), formal analysis (lead), writing – original draft (lead), writing – review and editing (lead). **Tommaso Nuzzo:** data curation (equal), investigation (equal). **Noemi Brolatti:** investigation (equal). **Maria Egle De Stefano:** data curation (equal), writing – review and editing (equal). **Enza Maria Valente:** data curation (equal), writing – review and editing (equal). **Francesco Errico:** data curation (equal), writing – review and editing (equal). **Claudio Bruno:** conceptualization (lead), data curation (equal), formal analysis (supporting), writing – review and editing (equal). **Alessandro Usiello:** conceptualization (lead), data curation (lead), formal analysis (equal), writing – review and editing (lead).

## Ethics Statement

This study was approved by the local ethics committee (Study protocol: COMETA‐DMD, 437/2023—dB id 13,411). Prior to their inclusion in the study, all potential participants and their guardians provided assent and consent, respectively. The study was conducted in accordance with the 1964 Declaration of Helsinki and its subsequent amendments.

## Consent

The authors have nothing to report.

## Conflicts of Interest

The authors declare no conflicts of interest.

## Supporting information


Appendix S1.

## Data Availability

The data that support the findings of this study are available from the corresponding author upon reasonable request.
