# Supplementary material for: D‐ and L‐Amino Acid Blood Concentrations Are Affected in Children With Duchenne Muscular Dystrophy
Source: J Cell Mol Med. 2025 May 6;29(9):e70495. doi: 10.1111/jcmm.70495 (PMC12055753; doi:10.1111/jcmm.70495)
Supplement: Supplementary file 1 — Appendix S1. [file JCMM-29-e70495-s001.docx]

**D- and L-amino acid blood concentrations are affected**

**in children with Duchenne muscular dystrophy**

Martina Garofalo, Chiara Panicucci, Alberto Imarisio, Tommaso Nuzzo, Noemi Brolatti, Maria Egle De Stefano, Enza Maria Valente, Francesco Errico, Claudio Bruno, Alessandro Usiello

**Supplementary Tables 1-4**

**Supplementary Table 1.** Control participants included in the study.

| Subject number | Age at collection (y) | Sex | Diagnosis |
| --- | --- | --- | --- |
| 1 | 12.7 | M | Constitutional growth delay |
| 2 | 13.8 | M | Constitutional growth delay |
| 3 | 24.8 | M | Klinefelter syndrome |
| 4 | 13.8 | M | Constitutional growth delay |
| 5 | 3.8 | M | Joint hyperlaxity |
| 6 | 6.0 | M | Mild psychomotor retardation |
| 7 | 4.1 | M | Neurofibromatosis type 1 |
| 8 | 5.5 | M | Previous infectious myositis |
| 9 | 15.7 | M | Constitutional growth delay |
| 10 | 8.6 | M | Compensated congenital hypothyroidism |
| 11 | 13.8 | M | Mild cognitive delay |
| 12 | 19.7 | M | Joint hyperlaxity |
| 13 | 6.8 | M | Unconfirmed celiac disease |
| 14 | 8.1 | M | Joint hyperlaxity |
| 15 | 15.3 | M | Puberal gynecomastia |
| 16 | 10.5 | M | Joint hyperlaxity |
| 17 | 14.5 | M | Constitutional growth delay |
| 18 | 12.1 | M | Constitutional growth delay |
| 19 | 3.1 | M | Constitutional growth delay |
| 20 | 15.2 | M | Constitutional growth delay |
| 21 | 6.3 | M | Polyautoimmune syndrome (alopecia totalis, chronic lymphocitic thyroiditis, celiac disease, suspected polyendocrinopathy-candidiasis-ectodermal dystrophy (APECED)) |
| 22 | 5.9 | M | Neurofibromatosis type 1 |
| 23 | 3.7 | M | Constitutional growth delay |
| 24 | 9.0 | M | Constitutional growth delay |

**Supplementary Table 2.** Comparison of serum amino acid levels between DMD patients treated or non-treated with deflazacort at blood sampling. Data are shown as median (IQR).

|  | **Non-treated (n = 12)** | **Treated (n = 17)** | **p^a^** |
| --- | --- | --- | --- |
| **D-aspartate (μM)** | 0.4 (0.3-0.5) | 0.3 (0.3-0.4) | 0.288 |
| **L-Aspartate (μM)** | 9.1 (7.9-13.5) | 8.5 (7.4-12.1) | 0.626 |
| **D-/Total aspartate (%)** | 4.2 (3.0-6.7) | 3.8 (2.9-6.0) | 0.859 |
| **L-asparagine (μM)** | 26.4 (23.9-28.4) | 27.8 (23.9-28.4) | 0.425 |
| **L-glutamate (μM)** | 28.0 (23.8-35.1) | 29.0 (21.9-37.5) | 0.690 |
| **L-glutamine (μM)** | 311.6 (265.8-337.3) | 307.6 (266.4-340.8) | 0.929 |
| **L-glutamine/L-glutamate** | 11.4 (9.1-13.1) | 11.0 (7.0-14.0) | 1.000 |
| **D-serine (μM)** | 0.7 (0.6-1.0) | 0.8 (0.5-1.0) | 0.757 |
| **L-serine (μM)** | 93.3 (74.6-116.1) | 95.5 (81.3-120.1) | 0.790 |
| **D-/Total serine (%)** | 0.8 (0.7-0.9) | 0.8 (0.6-0.9) | 1.000 |
| **Glycine (μM)** | 145.2 (130.0-167.5) | 159.8 (124.8-221.9) | 0.723 |
| **Glycine/L-serine** | 1.6 (1.2-2.1) | 1.6 (1.3-2.1) | 0.894 |

^a^ Mann-Whitney U test

**Supplementary Table 3.** Comparison of serum amino acid levels between ambulant and wheelchair-bound DMD patients. Data are shown as median (IQR).

|  | **Ambulant (n = 22)** | **Not-ambulant (n = 7)** | **p^a^** |
| --- | --- | --- | --- |
| **D-aspartate (μM)** | 0.4 (0.3-0.5) | 0.4 (0.3-0.5) | 0.610 |
| **L-Aspartate (μM)** | 8.4 (7.4-9.2) | 14.3 (10.1-14.7) | **0.001** |
| **D-/Total aspartate (%)** | 4.5 (3.4-6.2) | 3.4 (2.2-4.1) | 0.110 |
| **L-asparagine (μM)** | 27.3 (23.4-29.1) | 27.5 (24.4-29.6) | 0.980 |
| **L-glutamate (μM)** | 27.4 (21.0-34.4) | 34.6 (36.3-55.7) | **0.042** |
| **L-glutamine (μM)** | 307.3 (259.6-329.0) | 327.1 (280.1-383.6) | 0.165 |
| **L-glutamine/L-glutamate** | 12.6 (8.6-14.1) | 10.6 (6.2-11.0) | **0.042** |
| **D-serine (μM)** | 0.7 (0.5-1.0) | 0.8 (0.6-1.0) | 0.709 |
| **L-serine (μM)** | 88.5 (72.5-107.8) | 117.3 (110.3-125.4) | **0.009** |
| **D-/Total serine (%)** | 0.7 (0.9-1.0) | 0.6 (0.5-0.8) | **0.018** |
| **Glycine (μM)** | 141.6 (124.3- 174.7) | 206.3 (137.1-328.7) | **0.032** |
| **Glycine/L-serine** | 1.6 (1.4-2.0) | 1.8 (1.2-3.2) | 0.709 |

^a^ Mann-Whitney U test

**Supplementary Table 4.** Comparison of serum amino acid levels between DMD patients with normal and impaired cognition. Data are shown as median (IQR).

|  | **Normal cognition (n = 19)** | **Impaired cognition (n = 10)** | **p^a^** |
| --- | --- | --- | --- |
| **D-aspartate (μM)** | 0.4 (0.3-0.5) | 0.4 (0.3-0.5) | 0.630 |
| **L-Aspartate (μM)** | 9.1 (7.7-14.1) | 8.1 (6.9-10.5) | 0.215 |
| **D-/Total aspartate (%)** | 3.8 (2.9-6.0) | 4.6 (3.2-6.9) | 0.435 |
| **L-asparagine (μM)** | 27.7 (24.8-29.2) | 26.4 (23.3-29.1) | 0.680 |
| **L-glutamate (μM)** | 29.0 (23.6-34.6) | 30.0 (22.0-41.9) | 0.819 |
| **L-glutamine (μM)** | 311.8 (280.2-341.6) | 301.0 (252.5-320.3) | 0.271 |
| **L-glutamine/L-glutamate** | 11.0 (8.6-12.9) | 10.3 (6.7-13.4) | 0.614 |
| **D-serine (μM)** | 0.8 (0.6-1.0) | 0.7 (0.5-0.8) | 0.491 |
| **L-serine (μM)** | 96.4 (85.3-117.3) | 92.7 (71.8-118.8) | 0.748 |
| **D-/Total serine (%)** | 0.8 (0.6-0.9) | 0.7 (0.6-0.9) | 0.383 |
| **Glycine (μM)** | 155.1 (128.9-206.3) | 138.8 (121.5-248.8) | 0.582 |
| **Glycine/L-serine** | 1.6 (1.4-1.9) | 1.7 (1.1-2.2) | 0.963 |

^a^ Mann-Whitney U test
